# Correlation between increased atrial expression of genes related to fatty acid metabolism and autophagy in patients with chronic atrial fibrillation

**DOI:** 10.1371/journal.pone.0224713

**Published:** 2020-04-21

**Authors:** Yasushige Shingu, Shingo Takada, Takashi Yokota, Ryosuke Shirakawa, Akira Yamada, Tomonori Ooka, Hiroki Katoh, Suguru Kubota, Yoshiro Matsui

**Affiliations:** 1 Department of Cardiovascular and Thoracic Surgery, Faculty of Medicine and Graduate School of Medicine, Hokkaido University, Sapporo, Japan; 2 Department of Cardiovascular Medicine, Faculty of Medicine and Graduate School of Medicine, Hokkaido University, Sapporo, Japan; 3 Department of Cardiovascular Surgery, Teine Keijinkai Hospital, Sapporo, Japan; 4 Emergency and Critical Care Center, Hokkaido University Hospital, Sapporo, Japan; Indiana University, UNITED STATES

## Abstract

Atrial metabolic disturbance contributes to the onset and development of atrial fibrillation (AF). Autophagy plays a role in maintaining the cellular energy balance. We examined whether atrial gene expressions related to fatty acid metabolism and autophagy are altered in chronic AF and whether they are related to each other. Right atrial tissue was obtained during heart surgery from 51 patients with sinus rhythm (SR, n = 38) or chronic AF (n = 13). Preoperative fasting serum free-fatty-acid levels were significantly higher in the AF patients. The atrial gene expression of fatty acid binding protein 3 (*FABP3*), which is involved in the cells' fatty acid uptake and intracellular fatty acid transport, was significantly increased in AF patients compared to SR patients; in the SR patients it was positively correlated with the right atrial diameter and intra-atrial electromechanical delay (EMD), parameters of structural and electrical atrial remodeling that were evaluated by an echocardiography. In contrast, the two groups' atrial contents of diacylglycerol (DAG), a toxic fatty acid metabolite, were comparable. Importantly, the atrial gene expression of microtubule-associated protein light chain 3 (*LC3*) was significantly increased in AF patients, and autophagy-related genes including *LC3* were positively correlated with the atrial expression of *FABP3*. In conclusion, in chronic AF patients, the atrial expression of *FABP3* was upregulated in association with autophagy-related genes without altered atrial DAG content. Our findings may support the hypothesis that dysregulated cardiac fatty acid metabolism contributes to the progression of AF and induction of autophagy has a cardioprotective effect against cardiac lipotoxicity in chronic AF.

## Introduction

Atrial fibrillation (AF) is the most common cardiac arrhythmia, and its presence is associated with increased risks of death, heart failure, and stroke [1–3]. With the recent increase in prevalence of AF, the prevention of AF is important not only for public health but also to reduce the associated economic burden [4]. The risk factors for AF are diverse, including higher serum levels of free fatty acids (FFAs), obesity, hypertension, inflammation, and oxidative stress [5–7]. The mechanisms underlying the onset and development of AF have not been fully elucidated.

The pathophysiology of AF is complex and involves electrical, structural, contractile, and neurohormonal remodeling [8, 9]; metabolic disturbance in the atrial cardiac muscle is a recent focus of AF research, as the heart has a very high energy demand due to its organ-specific feature involving the constant activation of mitochondrial oxidative phosphorylation. In particular, fatty acids are the major fuel for the heart; their use depends on their uptake into the cells, transport from the cytosol into the mitochondria, and β-oxidation in the mitochondria. Prior research has shown that an elevated level of circulating FFAs is a strong risk factor for AF and AF-related stroke [6, 10] and can be a trigger of cardiac lipotoxicity, which is defined as the excess accumulation of toxic fatty acid metabolites such as diacylglycerol (DAG) in the heart. This may occur when the influx of FFAs exceeds the intracellular fatty acid oxidation, which leads to cardiac dysfunction, cardiac remodeling, and arrhythmias [11]. However, it is still unclear how metabolic disturbances including abnormal fatty acid metabolism contribute to the development of AF.

Autophagy, the process of the degradation of intracellular components (e.g., proteins) in lysosomes, plays an important role in cellular homeostasis via cellular quality control. Autophagy was also shown to contribute to the cellular energy balance, in particular through a mechanism of fatty acid metabolism termed "lipophagy" (the degradation of excess lipids by autophagy) and the degradation of lipid stores in the cells [12]. Accordingly, autophagy may regulate fatty acid metabolism in cardiomyocytes. Alterations of the autophagy in the atrial muscles of patients with persistent AF [13, 14] or post-operative AF have been reported [15]. Although it is still controversial whether the induction of autophagy has a cardioprotective or detrimental effect in AF, it is possible that autophagy is involved in metabolic remodeling in the atrium in chronic AF patients.

We conducted the present study to determine: (1) whether the expression of genes related to fatty acid metabolism and autophagy are altered in the atria of patients with chronic AF, and (2) whether changes in these gene expression patterns are correlated with each other. We used human atrial tissue excised from patients during cardiac surgery, and our findings provide new insight into the pathophysiology of AF, focusing on fatty acid metabolism and autophagy in the human atrium.

## Materials and methods

### Patients

This study was conducted at Hokkaido University Hospital and Teine Keijinkai Hospital and included 51 consecutive patients: 38 with sinus rhythm (SR) and 13 with chronic AF who underwent cardiovascular surgery between 2013 and 2019 at either of these hospitals. All of the patients were Japanese. The patients with SR in the present series partly overlap with those of our recently published report [16].

After the establishment of a cardiopulmonary bypass (10 min after the infusion of heparin 300 IU/kg), right atrial myocardial tissue (approx. 100 mm^2^) was excised from the right atrial incision site or the insertion point of a drainage cannula. The tissue was frozen and stored at −80°C until analysis.

Type 2 diabetes was defined as a fasting glucose level ≥7.0 mmol/L and/or taking antidiabetic medications. Coronary artery disease was evaluated by coronary angiography, and stenosis ≥75% was defined as significant; a patient with a history of percutaneous coronary intervention was also regarded as having coronary artery disease.

The study protocol was approved by the Ethics Committees of Hokkaido University Hospital and Teine Keijinkai Hospital and performed according to the Declaration of Helsinki. Written informed consent was obtained from each patient before the surgery. This study was registered in the UMIN Clinical Trials Registry: UMIN000012405 and UMIN000018137.

### Transthoracic echocardiography

A Vivid Seven system (GE/Vingmed, Milwaukee, WI) with an M3S (2.5–3.5 MHz) transducer, an Aplio system (Toshiba Medical Systems, Tokyo, Japan) with a 2.5-MHz transducer, or a Philips system (Philips Ultrasound, Bothell, WA) with a 2.5-MHz transducer was used for the pre-operative echocardiography. The left ventricular and atrial diameters were measured from the parasternal long-axis view. The right atrial diameter (the largest minor-axis diameter in the four-chamber view) was obtained at end-systole [17].

The electromechanical delay (EMD) of the right atrium was assessed in all but one of the SR patients as a time interval (T1) from the beginning of the P-wave on surface ECG to the beginning of the late diastolic wave (A') of the tricuspid annulus [17]. T2 was the time interval from the beginning of the P-wave on the surface ECG to the A' of the septal mitral annulus. The intra-atrial EMD was the interval from T1 to T2, expressed as T2-T1 (msec). The left ventricular ejection fraction (LVEF) was measured using the biplane method of disks. The mitral regurgitation grade was defined by the regurgitation jet area-to-left atrium ratio (mild, <20%; moderate, 20%–40%; severe, >40%).

Tricuspid regurgitation was graded using the regurgitation jet area (mild, <5 cm^2^; moderate, 5–10 cm^2^; severe, >10 cm^2^). Aortic stenosis was defined using the valve area (mild, >1.5 cm^2^; moderate, 1.0–1.5 cm^2^; severe, <1.0 cm^2^). Aortic regurgitation was determined using a combination of the jet width/outflow tract, the pressure half-time, and the diastolic reverse flow at the abdominal aorta (mild, moderate, severe) [18].

### Blood biochemistry

Blood was collected from each patient early in the morning after a 10-hr fast within 2 days before surgery. The following biochemical values were analysed by the methods shown in **S1 Table**: the blood glucose, hemoglobin A1c, insulin, FFA, total cholesterol, triglycerides, and B-type natriuretic peptide (BNP).

### The atrial expression of genes related to fatty acid metabolism and autophagy

The atrial expression of the following genes related to fatty acid metabolism and autophagy was determined by reverse transcription-polymerase chain reaction (RT-PCR): cluster of differentiation 36/fatty acid translocase (*CD36*), carnitine palmitoyltransferase 1B (*CPT1B*), fatty acid-binding protein 3 (*FABP3*), autophagy-related gene 5 (*ATG5*), Unc-51-like kinase 1 (*ULK1*), Beclin-1 (*BCLN1*), and microtubule-associated protein light chain 3 (*LC3*). Myocardial total RNA was isolated from frozen tissue using a High Pure RNA Tissue Kit (Roche, Penzberg, Germany) and was then reversely transcribed into cDNA using a Transcriptor First Strand cDNA Synthesis Kit (Roche).

The RT-PCR was performed using FastStart Essential DNA Probes Master (Roche) and the Real-time Ready Assay (Roche Assay ID: *CD36*, 144833; *CPT1B*, 126501; *FABP3*, 118811; *ATG5*, 125999; *ULK1*, 109914; *BCLN1*, 100115; *LC3*, 144582; *TBP*, 143707). Detailed information about probes and primers is shown in **S2 Table**. PCR amplification was then performed with a reaction volume of 20 μL using a LightCycler Nano (Roche) under the conditions specified by the manufacturer. After the initial denaturation and activation of the enzyme for 10 min at 95°C, 45 cycles of denaturation at 95°C for 10 sec and annealing and extension at 60°C for 30 sec were performed. *TBP* (TATA-binding protein) was used as a reference gene, as we confirmed that TBP is not influenced by the occurrence of AF.

### The atrial protein expression level of autophagy

We measured atrial protein expression level of LC3-II, an autophagosome marker, by Western blot analysis. A semi-dry Western blot apparatus (Mini-PROTEAN Tetra Cell, BIO-RAD, CA) detected the conversion from LC3-I (cytosolic form) to LC3-II (membrane-bound lipidated form). The amount of LC3-II protein reflects the number of autophagosomes. After sodium dodecyl sulfate-polyacrylamide gel electrophoresis (12% Mini-PROTEANTGXTM, BIO-RAD, CA), the proteins were blotted to a polyvinyldine deflouride membrane and incubated with primary (Anti-LC3B, Abcam, Cambridge, UK) and secondary antibodies (Anti-rabbit IgG, Cell Signaling, MA). The bands were quantified by chemiluminescence using JustTLC (Sweday, Sodra Sandby, Sweden). The membranes were dyed with naphthol blue black solution to normalize the band intensity. Due to the lack of atrial muscle samples, the protein expression level of LC3-II was measured in three patients with SR and four patients with AF.

### The atrial enzymatic activities of the mitochondrial TCA cycle and fatty acid β-oxidation

We spectrophotometrically determined the activity levels of both citrate synthase (CS), a key enzyme in the tricarboxylic acid (TCA) cycle, and β-hydroxyacyl CoA dehydrogenase (β-HAD), a key enzyme in fatty acid β-oxidation, in the myocardial samples as described [19]. Due to the lack of atrial muscle samples, these enzymatic activities were measured in 20 patients with SR and eight patients with AF.

### The atrial DAG content

Heart tissue was homogenized in 1.5 mL of methanol, followed by mixing with 2.25 mL of 1 M NaCl and 2.5 mL of chloroform. The mixture was centrifuged at 1,500 g for 10 min at 4°C, and the organic phase was dried. DAG was then detected with a DAG assay kit (Cell Biolabs, San Diego, CA) following the manufacturer's instructions. Due to the lack of atrial muscle samples, the atrial DAG content was measured in five patients with SR and six patients with AF.

### Statistical analyses

Values are presented as the mean ± standard deviation (SD) or the median (interquartile range [IQR]) as appropriate. We used Student's t-test for continuous variables that are normally distributed and the Mann-Whitney U-test for other continuous variables. The chi-square test or Fisher's exact test were used for categorical variables. We conducted a Pearson's correlation analysis to determine linear relationships between continuous variables. The statistical analyses were performed using GraphPad Prism ver. 8 (GraphPad Software, San Diego, CA), and significance was defined as p<0.05.

## Results

### Patient characteristics

The characteristics of the SR and AF patients are summarized in **Table 1**. The median duration of AF was 12 years (range 1–16 years). Except for the use of medications, all parameters including cardiovascular risk factors were comparable between the two groups. The surgical procedures performed after the excision of atrial specimens included aortic valve replacement in 30 cases, total arch replacement in eight cases, coronary artery bypass grafting in 10 cases, aortic root replacement in six cases, and mitral valve repair in 12 cases. Multiple procedures were performed in some patients.

**Table 1:** Characteristics of the SR and AF patients.

	SR (n = 38)	AF (n = 13)	p-value
Age, yrs	70 ± 13	69 ± 9	0.775
Male	18 (47%)	8 (62%)	0.378
BMI, kg/m^2^	22.8 ± 3.4	24.8 ± 4.3	0.097
Heart rate, bpm	67 ± 10	73 ± 13	0.134
Systolic blood pressure, mm Hg	118 ± 19	115 ± 18	0.643
Diastolic blood pressure, mm Hg	60 ± 12	68 ± 13	0.051
Diabetes mellitus	7 (18%)	2 (15%)	0.804
Coronary artery disease	13 (34%)	2 (15%)	0.198
Medications			
Diuretics	18 (47%)	13 (100%)	0.001
β-blockers	14 (37%)	9 (69%)	0.043
Statins	18 (47%)	4 (31%)	0.297
Total cholesterol, mmol/L	4.7 ± 0.9	4.4 ± 0.7	0.357
Triglycerides, mmol/L	1.4 ± 0.7	1.0 ± 0.5	0.052
Fasting blood glucose, mmol/L	5.5 (0.8)	5.7 (0.7)	0.802
Insulin, μU/mL	5.0 (4.0)	5.8 (8.9)	0.612
HbA1c, %	5.7 ± 0.8	5.9 ± 0.6	0.324
BNP, pg/mL	79 (270)	249 (321)	0.060

Data are mean±SD, median (interquartile range), or n (%). AF, atrial fibrillation; BMI, body mass index; BNP, B-type natriuretic peptide; HbA1c, hemoglobin A1c; SR, sinus rhythm.

### Transthoracic echocardiography

As shown in **Table 2**, the diameters of the left and the right atria were significantly increased in the AF group. The left ventricular size (i.e., left ventricular end-diastolic diameter [LVDd]) and the left ventricular systolic function (i.e., the LVEF) were comparable between the two groups. Regarding valvular heart disease, the grade of mitral regurgitation was higher in the AF group than in the SR group.

**Table 2:** Echocardiographic parameters of the SR and AF patients.

	SR (n = 38)	AF (n = 13)	p-value
LVDd, mm	48 (20)	59 (17)	0.209
LVDs, mm	31 (22)	41 (20)	0.132
LVEF, %	59 ± 14	54 ± 14	0.268
Left atrial diameter, mm	41 ± 7	53 ± 9	<0.001
Right atrial diameter, mm	34 ± 6	42 ± 9	0.007
Aortic stenosis:			0.130
Mild	1 (3%)	0 (0%)	
Moderate	1 (3%)	1 (8%)	
Severe	15 (39%)	1 (8%)	
Aortic regurgitation:			0.306
Mild	18 (47%)	4 (31%)	
Moderate	4 (11%)	2 (15%)	
Severe	10 (26%)	2 (15%)	
Mitral regurgitation:			0.002
Mild	29 (76%)	5 (38%)	
Moderate	2 (5%)	5 (38%)	
Severe	2 (5%)	3 (23%)	
Tricuspid regurgitation:			0.133
Mild	26 (68%)	6 (46%)	
Moderate	3 (78%)	3 (23%)	
Severe	0 (0%)	1 (8%)	

Data are mean±SD, median (interquartile range), or n (%). AF, atrial fibrillation; LVDd, left ventricular end-diastolic diameter; LVDs, left ventricular end-systolic diameter; LVEF, left ventricular ejection fraction; SR, sinus rhythm.

### The serum levels of FFA and the atrial expression of genes related to fatty acid metabolism

**Fig 1:**
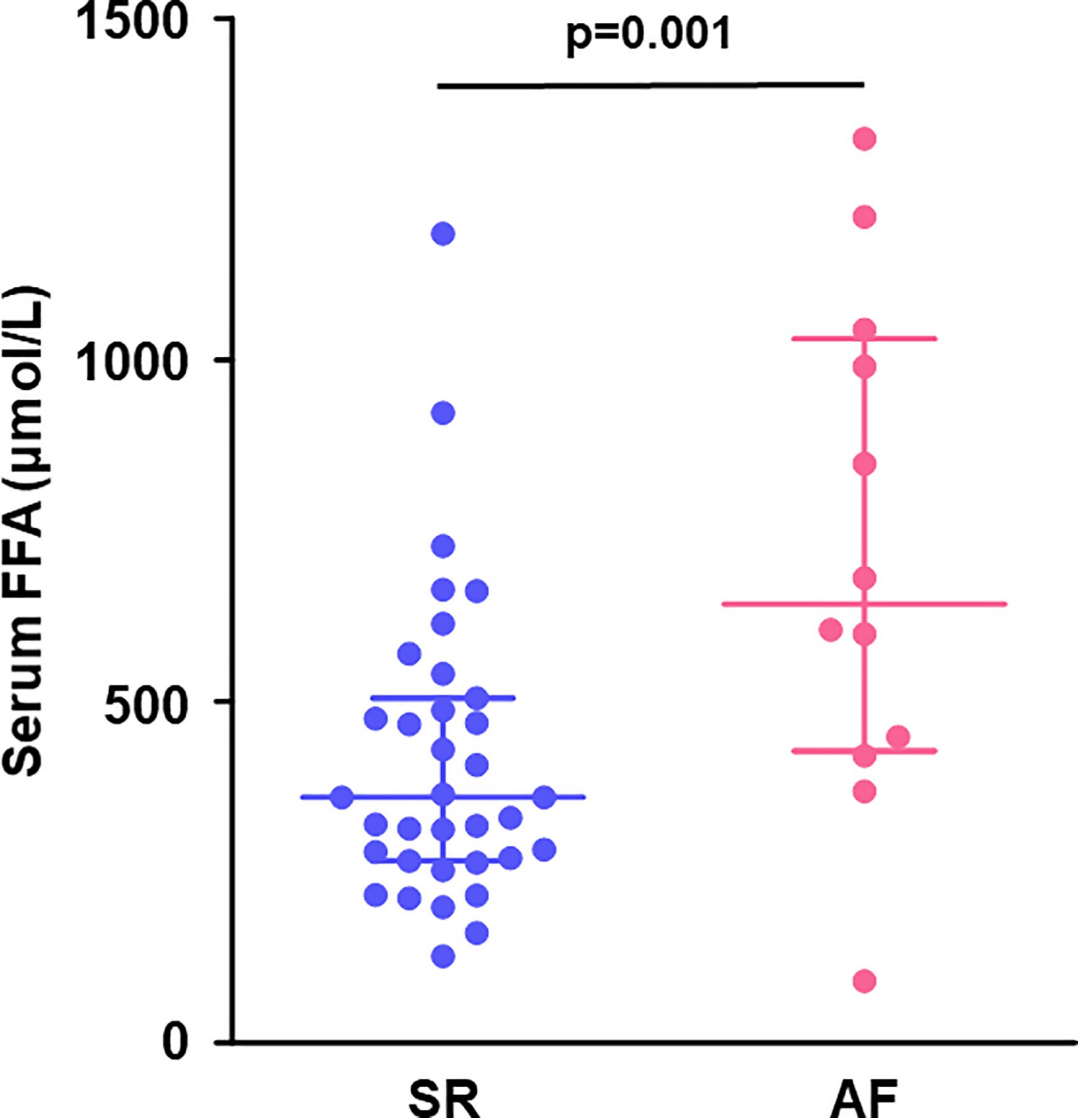
Preoperative levels of serum FFA. AF patients had a higher levels of serum FFA than SR patients. Lines indicate the median with the interquartile range in each group (SR, n = 35; AF, n = 12). AF, atrial fibrillation; FFA, free fatty acid; SR, sinus rhythm.

The serum FFA levels were significantly higher in the AF group than the SR group (719 ± 107 vs. 416 ± 37 μmol/L, p = 0.001, **Fig 1**).

**Fig 2:**
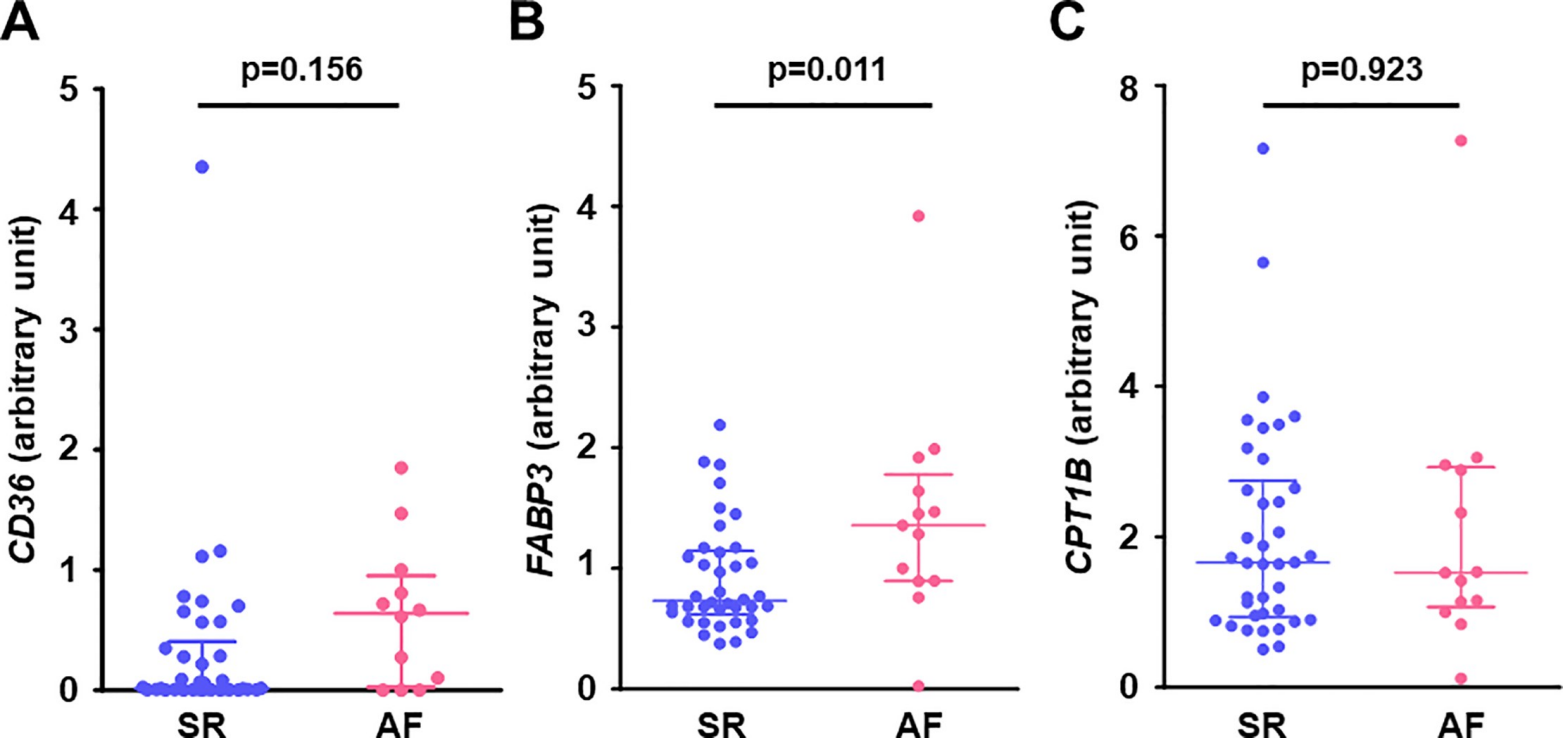
Gene expression related to fatty acid metabolism in the right atrial myocardium. (**A**) *CD36*, (**B**) *FABP3*, and (**C**) *CPT1B*. Atrial gene expression of *FABP3* (which facilitates fatty acid uptake into the cell and intracellular fatty acid transport) was significantly higher in the AF group than in the SR group, however, there was no difference in *CD36* (which facilitates fatty acid uptake across the plasma membrane) and *CPT1B* (which facilitates fatty acid transport into the mitochondria) between the groups. Lines indicate the median with interquartile range in each group (SR; n = 38 except for *CD36* [n = 37], AF; n = 13). *CD36*, cluster of differentiation 36 (fatty acid translocase); *CPT1B*, carnitine palmitoyltransferase 1B; *FABP3*, fatty acid binding protein 3.

The expressions of genes related to fatty acid metabolism in the right atrial muscle are shown in **Fig 2**. The gene expression of *FABP3*, which facilitates fatty acid uptake into the cell and intracellular fatty acid transport, was significantly increased in the AF group (**Fig 2B**), but there was no significant difference between the two groups in the atrial expression of *CD36*, which facilitates fatty acid uptake across the plasma membrane, and *CPT1B*, which is located on the outer mitochondrial membrane for fatty acid transport into the mitochondria (**Fig 2A–2C**).

**Fig 3:**
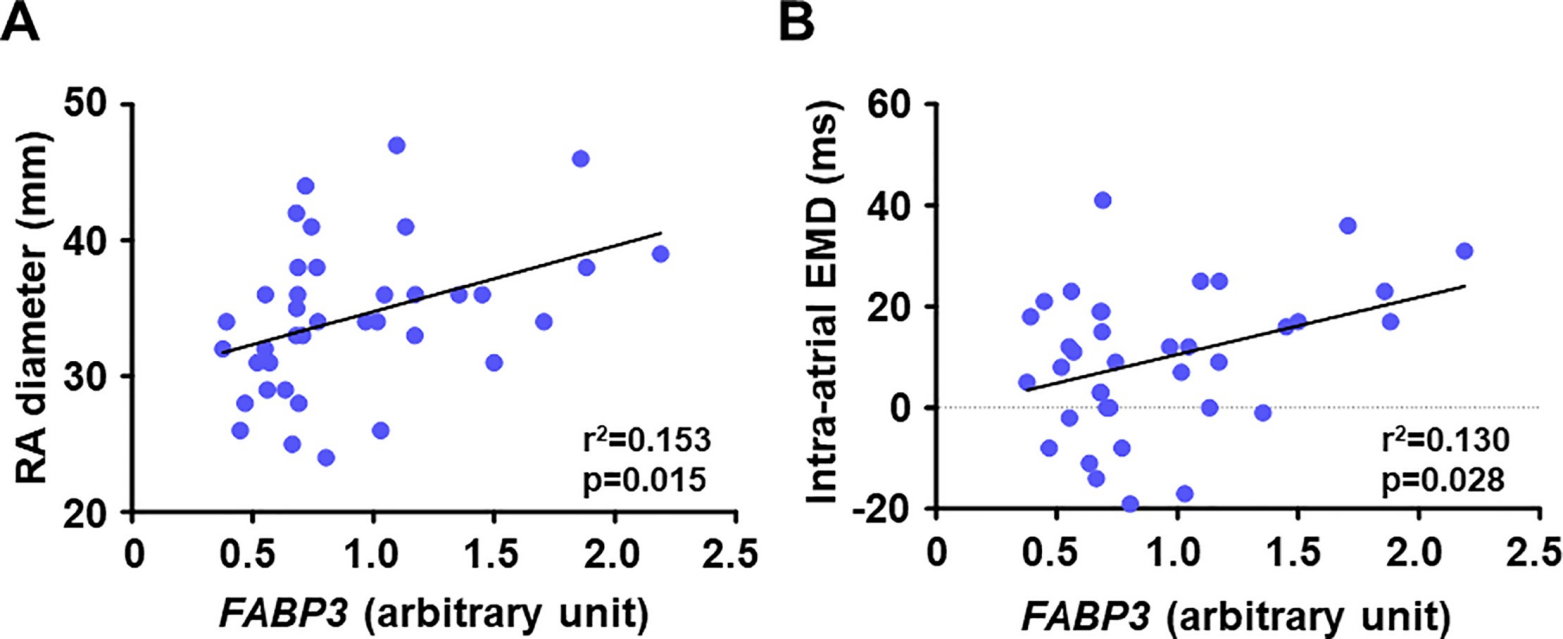
Association between gene expression levels of *FABP3* in the right atrial myocardium and parameters of atrial remodelling in patients with SR. (**A**) *FABP3* and RA diameter (n = 38) and (**B**) *FABP3* and intra-atrial EMD (n = 37). Atrial gene expression of *FABP3* was positively correlated with RA diameter (a marker of structural atrial remodeling) and intra-atrial EMD (a marker of electrical atrial remodeling) in patients with SR. EMD, electromechanical delay; *FABP3*, fatty acid binding protein 3; RA, right atrium.

In SR patients, *FABP3* gene was positively correlated with the right atrial diameter (**Fig 3A**) and the intra-atrial EMD (**Fig 3B**), indicating that increased atrial gene expression related to intracellular fatty acid transport was associated with structural and electrical atrial remodeling.

### The atrial enzymatic activities of the mitochondrial TCA cycle and fatty acid β-oxidation

**Fig 4:**
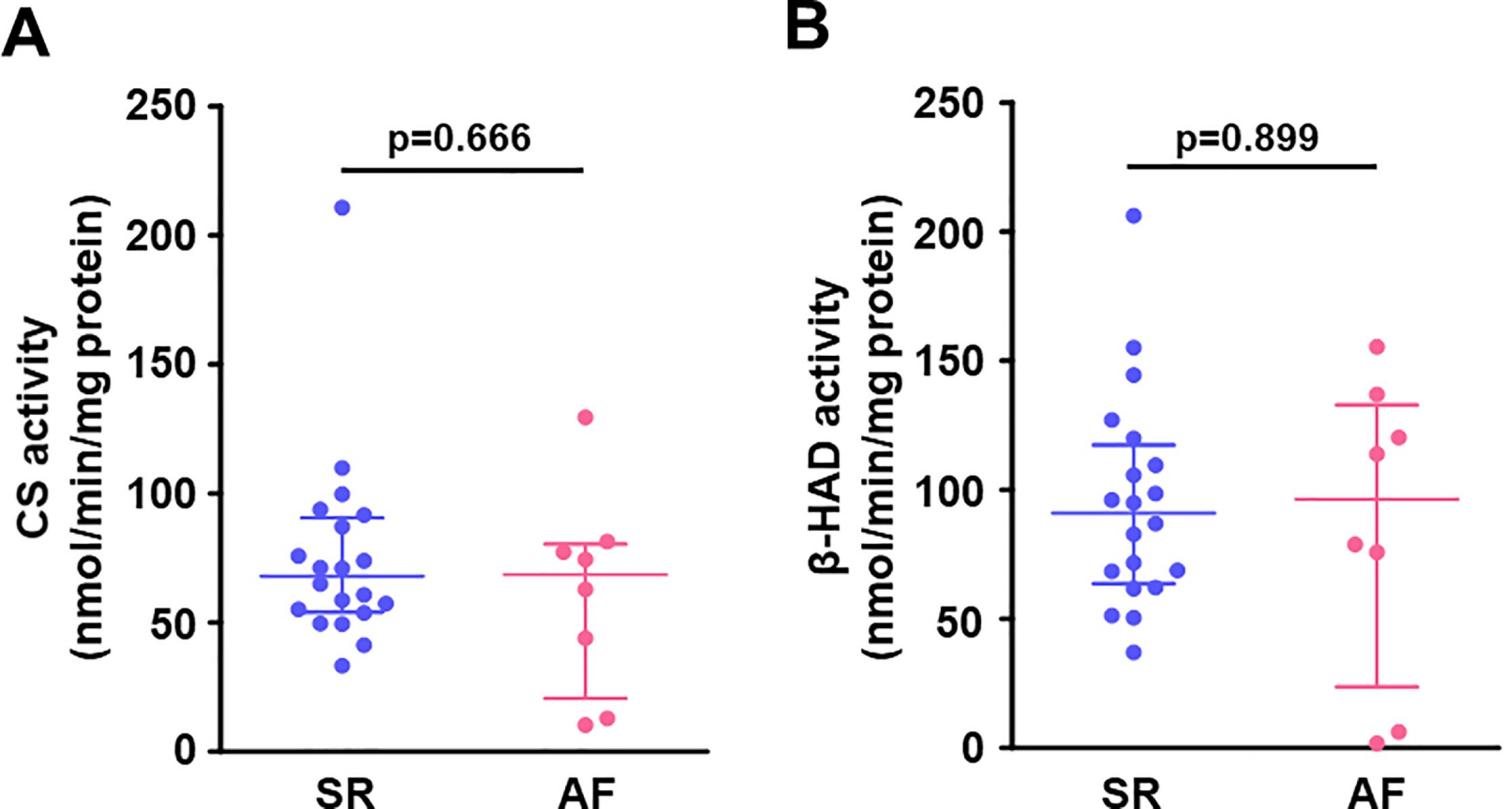
Enzymatic activities related to the mitochondrial TCA cycle and fatty acid β-oxidation in the right atrial myocardium. (**A**) CS activity and (**B**) β-HAD activity. There were no differences in activities of CS (a key enzyme in the mitochondrial TCA cycle) and β-HAD (a key enzyme in mitochondrial fatty acid β-oxidation) in the right atrium between the groups. Lines indicate the median with interquartile range in each group (SR, n = 20; AF, n = 8). β-HAD, β-hydroxyacyl CoA dehydrogenase; CS, citrate synthase.

**Fig 4** illustrates the enzymatic activities related to the mitochondrial fatty acid β-oxidation and TCA cycle in the right atrial muscle. The CS activity was comparable between the SR and AF groups (**Fig 4A**), as was the β-HAD activity (**Fig 4B**).

### The atrial DAG content

**Fig 5:**
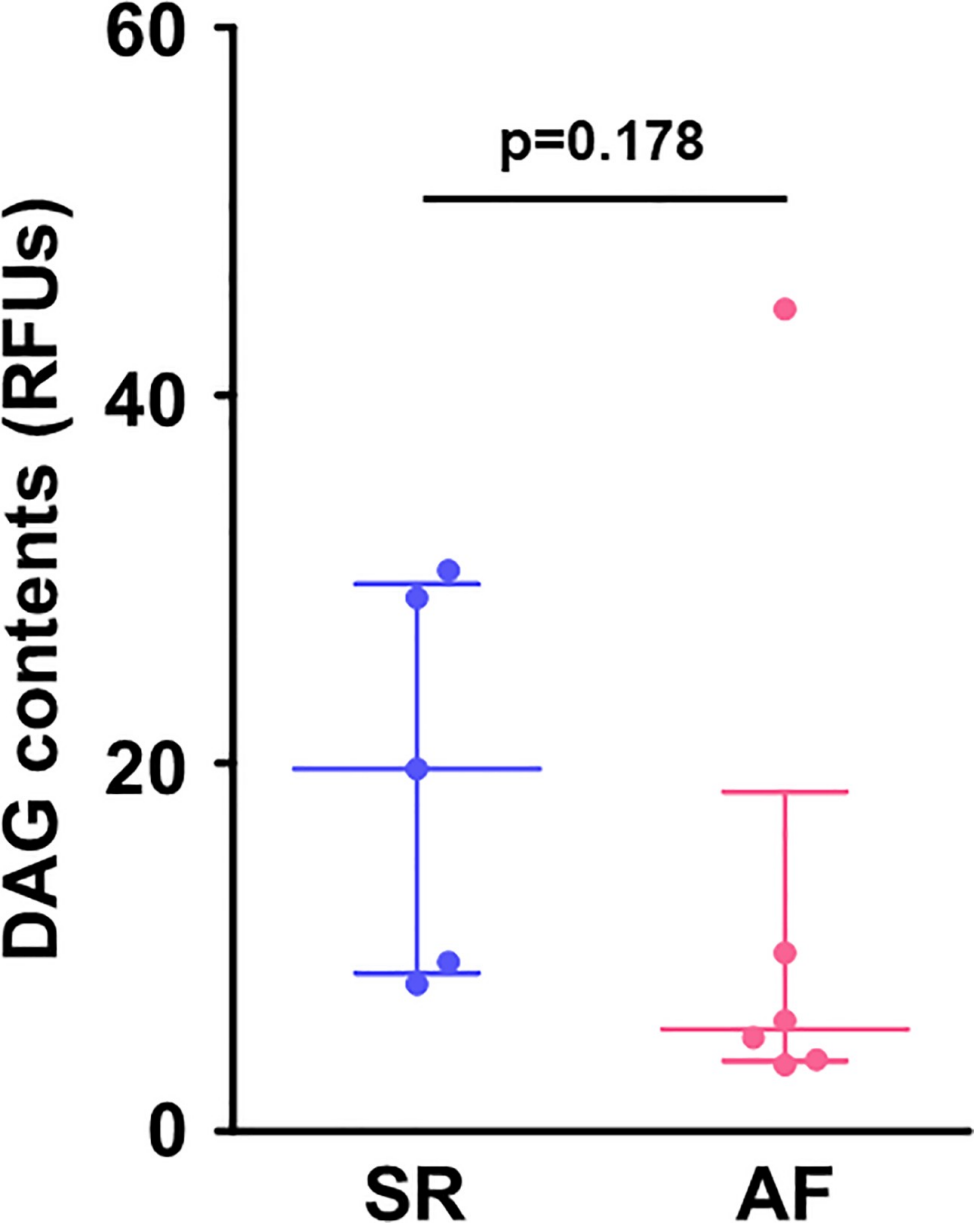
DAG contents in the right atrial myocardium. There was no difference in atrial contents of DAG (a toxic fatty acid metabolite) between the groups. Lines indicate the median with interquartile range in each group (SR, n = 5; AF, n = 6). DAG, diacylglycerol; RFUs, relative fluorescence units.

Despite the higher serum levels of FFA and the upregulated atrial gene expression of *FABP3* in the AF group, there was no significant difference in the atrial content of DAG, a major toxic fatty acid metabolite, between the SR and AF groups (**Fig 5**).

### The atrial expression of autophagy-related genes

**Fig 6:**
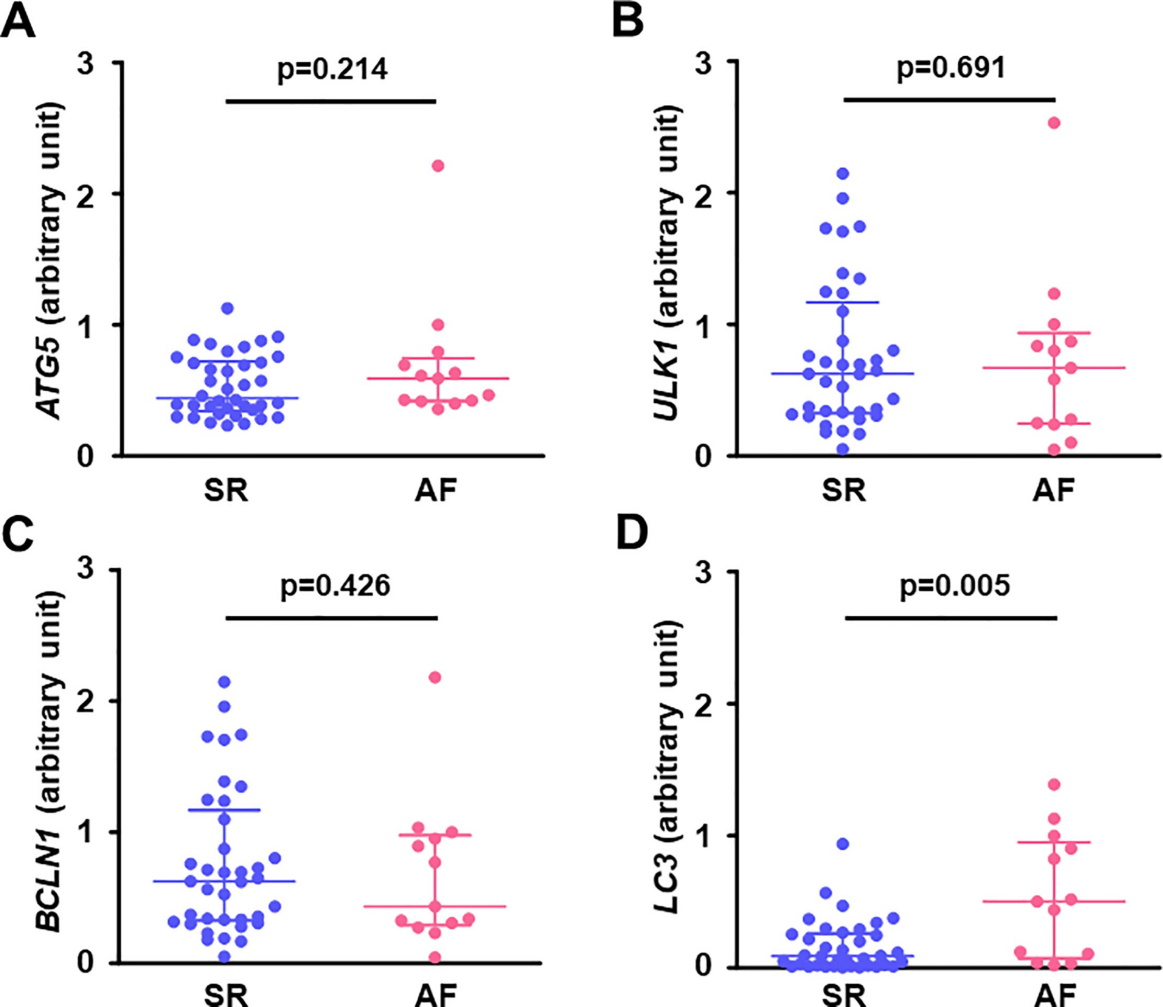
Gene expression related to autophagy in the right atrial myocardium. (**A**) *ATG5*, (**B**) *ULK1*, (**C**) *BCLN1*, and (**D**) *LC3*. Atrial gene expression of *LC3* was higher in the AF group than in the SR group, but there were no differences in atrial gene expressions of *ATG5*, *ULK1*, and *BCLN1* between the groups. Lines indicate the median with interquartile range in each group (SR, n = 37 except for *ATG5* [n = 38]; AF, n = 13). *ATG5*, autophagy-related gene 5; *BCLN1*, beclin-1; *LC3*, microtubule-associated protein light chain 3; *ULK1*, Unc-51-like kinase 1.

The gene expression of *LC3* in the right atrial muscle was significantly higher in the AF group than in the SR group (**Fig 6D**), but there was no significant difference in other autophagy-related genes including *ATG5*, *ULK1*, and *BCLN1* between the SR and AF groups (**Fig 6A–6C**).

### The linear relationship between the atrial expression of FABP3 gene and autophagy-related genes

**Fig 7:**
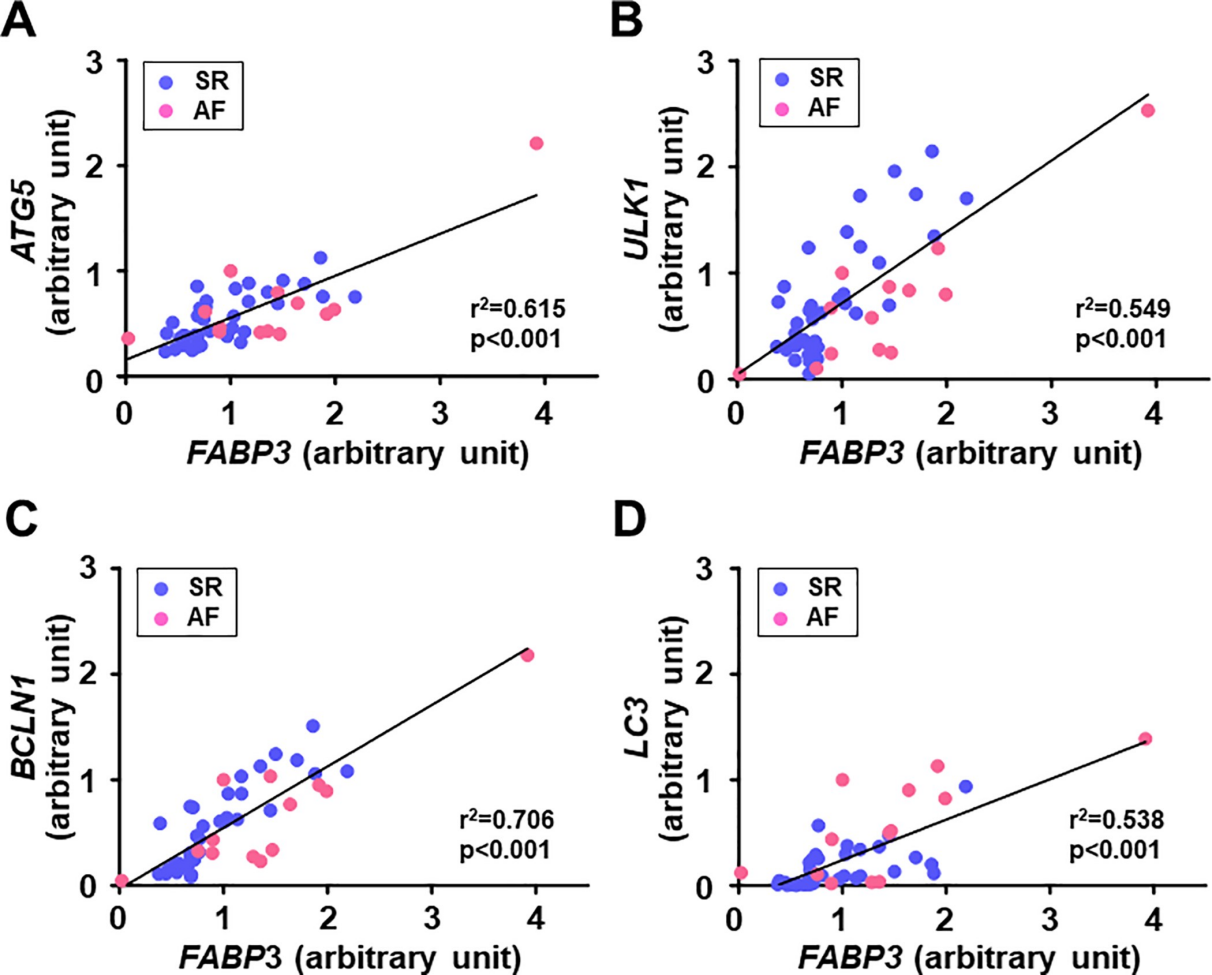
Association between the expression levels of *FABP3* gene and autophagy-related genes in the right atrial myocardium in patients with SR and AF. (**A**) *FABP3* and *ATG5* (SR; n = 13; AF, n = 38), (**B**) *FABP3* and *ULK1* (SR, n = 13; AF, n = 37), (**C**) *FABP3* and *BCLN1* (SR, n = 13; AF, n = 37), and (**D**) *FABP3* and *LC3* (SR, n = 13; AF, n = 37). Atrial gene expression of *FABP3* was positively correlated with atrial expression of genes related to autophagy including *LC3* in patients with SR and AF. *FABP3*, fatty acid binding protein 3. Other abbreviations are explained in the Fig 6 legend.

The gene expression of *FABP3* was positively correlated with autophagy-related genes including *LC3* gene in all patients (**Fig 7**). Similar correlations were observed between *FABP3* gene and autophagy-related genes even when they were analyzed separately for the SR group (*FABP3* and *ATG5*: r^2^ = 0.510, p<0.001; *FABP3* and *ULK1*: r^2^ = 0.622, p<0.001; *FABP3* and *BCLN1*: r^2^ = 0.740, p<0.001; *FABP3* and *LC3*: r^2^ = 0.384, p<0.001) and the AF group (*FABP3* and *ATG5*: r^2^ = 0.670, p<0.001; *FABP3* and *ULK1*: r^2^ = 0.790, p<0.001; *FABP3* and *BCLN1*: r^2^ = 0.768, p<0.001; *FABP3* and *LC3*: r^2^ = 0.534, p = 0.005).

### The atrial protein expression level of autophagy

**Fig 8:**
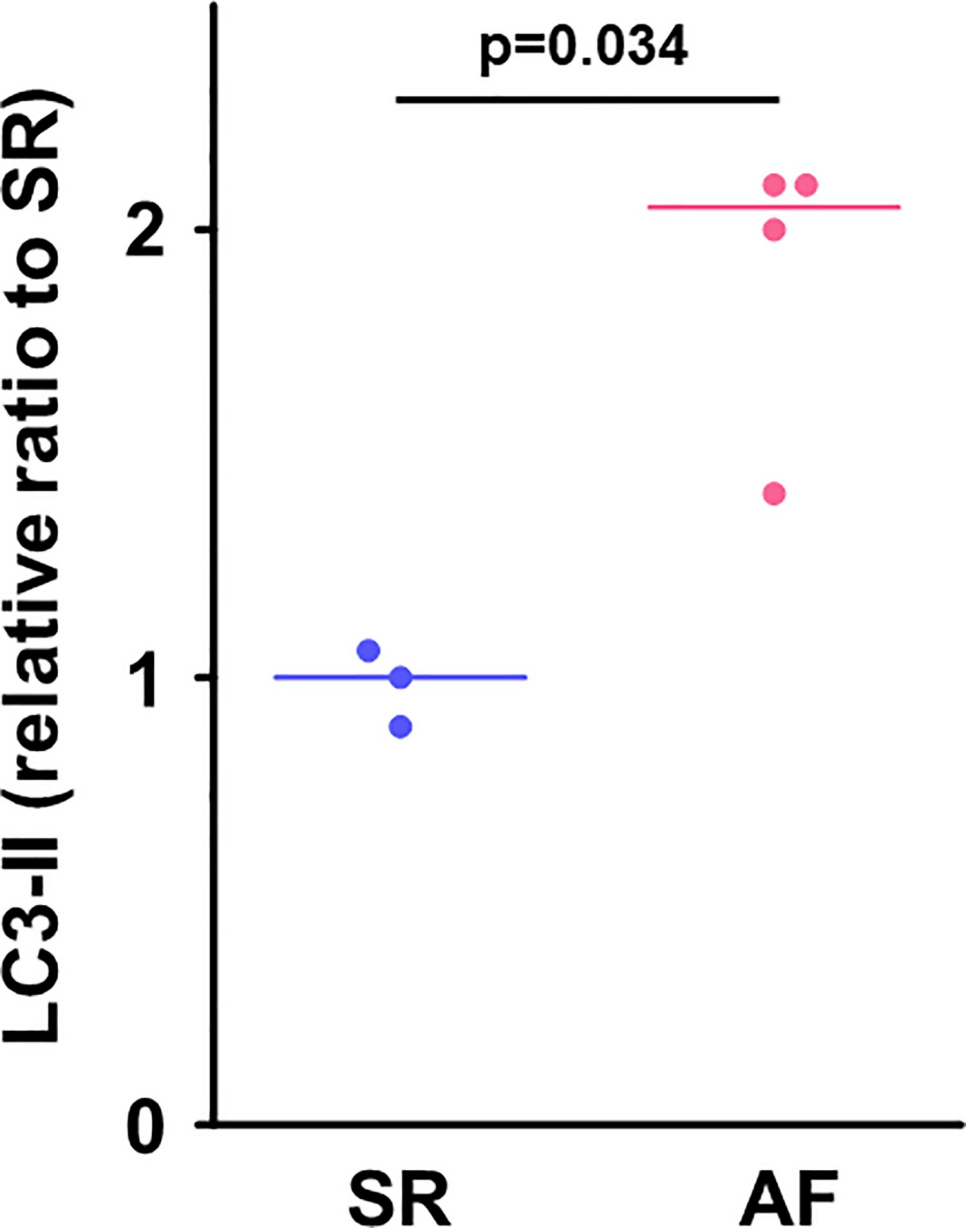
Protein expression level of LC3-II in the right atrial myocardium. Protein expression level of LC3-II, an autophagosome marker, in the right atrium was significantly increased in the AF group compared to the SR group. Lines indicate the median in each group (SR, n = 3; AF, n = 4). The unit expresses relative ratio to the median value of the SR group. LC3-II, microtubule-associated protein light chain 3-II.

In consistent with the increased gene expression of *LC3* in the AF group, the protein expression level of LC3-II in the right atrial muscle was higher in the AF group than in the SR group (**Fig 8**).

## Discussion

Our findings demonstrated that the atrial expression of a gene involved in fatty acid metabolism, *FABP3*, was upregulated in patients with chronic AF compared to the SR patients. In the SR patients, the increased atrial expression of *FABP3* was positively correlated with the right atrial diameter and the intra-atrial EMD, which are parameters of structural and electrical remodeling of the atrium. In contrast, there was no increase in the atrial content of DAG despite the increased atrial expression of *FABP3* and higher serum levels of FFA in patients with chronic AF. Intriguingly, the atrial expression of a gene related to autophagosome maturation, *LC3*, was increased in chronic AF patients, and autophagy-related genes including *LC3* were positively correlated with the atrial expression of *FABP3*. In consistent with the increased atrial gene expression of *LC3*, the atrial protein expression level of LC3-II, an autophagosome marker, was increased in patients with chronic AF. To the best of our knowledge, this is the first study showing that the atrial expression of *FABP3* gene is increased in association with autophagy-related genes in patients with chronic AF.

### The dysregulated fatty acid metabolism in the atrium of chronic AF patients

Compared to SR patients, chronic AF patients had higher serum FFA levels. The Cardiovascular Health Study has reported that an increase in the plasma levels of FFA by 200 μmol/L presents an 11% higher risk of AF occurrence even after adjustment for confounding risk factors including age, sex, race, physical activity, body mass index, coronary heart disease, congestive heart failure, smoking, alcohol use, log-C-reactive protein, diabetes mellitus, and hypertension in older adults [6]. Accordingly, elevated FFA levels can be an independent risk factor of AF.

The fatty acid-binding proteins (FABPs) reversibly bind to fatty acid and other lipophilic molecules. FABPs, which are located on the plasma membrane, facilitate fatty acid uptake into the cells, and intracellular FABPs transport fatty acid to other locations such as the nucleus and mitochondrion [20]. Among the 10 isoforms of FABPs distributed in various tissues in mammals, FABP3 is most predominantly expressed in the heart [21]. Here, we observed that the gene expression of *FABP3* in the right atrial muscle was enhanced in chronic AF patients, which may indicate increased fatty acid uptake into the cells and increased intracellular fatty acid transport in the atrial muscle in chronic AF. In contrast, the gene expression of *CPT-1B* (which facilitates fatty acid transport across the outer mitochondrial membrane), and β-HAD activity (an enzymatic activity of mitochondrial fatty acid β-oxidation) in the atrium were comparable between our AF and SR groups.

### The association of the atrial expression of FABP3 with structural and electrical atrial remodeling in SR patients

The results of our analyses revealed that the expression of *FABP3* in the right atrial muscle was positively correlated with the right atrium diameter and the intra-atrial EMD in SR patients. The intra-atrial EMD is the time delay from the electrical activation to the actual motion of the atrial myocardium, and a delayed intra-atrial EMD indicates excitation-contraction uncoupling in the atrium. Prolonged intra-atrial EMD after cardioversion was reported to predict AF recurrence in patients with persistent AF, and histopathological changes characterized by myocardial fibrosis in the atrium appear to be a major determinant of the prolonged intra-atrial EMD [22].

Boldt et al. revealed that the atrial expression of collagen type I is enhanced in patients with lone AF, indicating that the occurrence of AF can directly increase the expression of collagen type I and cause myocardial fibrosis in the atrial muscle [23]. Although we did not conduct histopathological evaluations, previous reports and our present findings raise the possibility that the dysregulation of atrial fatty acid metabolism is linked to structural and electrical atrial remodeling, which may contribute to a future onset or recurrence of AF. Here, we cannot conclude that there is a causal relationship between dysregulation of atrial fatty acid metabolism and atrial remodeling in chronic AF. Further studies with a larger sample size of chronic AF patients are needed to examine whether chronic or persistent AF might contribute to atrial remodeling via abnormal fatty acid metabolism in the atrium.

### Altered autophagy in the atrium in chronic AF

In 2012, Garcia et al. first reported impaired cardiac autophagy characterized by reduced LC3 processing (i.e., a reduced protein expression of LC3BⅡ) with an accumulation of lipofuscin deposit — a potential trigger of AF — in the atrial myocardium in patients with post-operative AF [15]. A pair of studies have shown that in chronic AF patients, the cardiac autophagy characterized by an increased protein expression of LC3BⅡ is induced in the atrial myocardium in association with AMPK or endoplasmic reticulum (ER) stress [13, 14]. Our present findings demonstrated that the atrial gene expression of *LC3* was upregulated and atrial protein expression of LC3-II was increased in patients with chronic AF. Taking these results together, we speculate that altered cardiac autophagy in the atrium may be involved in the progression of AF.

In addition, animal studies have shown that onset of ventricular fibrillation increases autophagy-associated proteins in the ventricle accompanied by myocardial damage [24–26], suggesting that arrhythmia itself may directly cause altered cardiac autophagy.

### Implications of the association between the atrial expression of FABP3 and autophagy

The intracellular lipid content is generally deteremined by an imbalance between the uptake and the utilization of fatty acid, and thus the increased atrial expression of *FABP3* that we observed might contribute to the accumulation of lipids including DAG in the atrial myocardium in chronic AF. However, we did not detect an accumulation of DAG in the atrium in our patients with chronic AF as was reported in another study [27]. Autophagy has been shown to play a role in the regulation of fatty acid metabolism via the degradation of excessive intracellular lipids, termed “lipophagy” [12]. Our findings of an association between the atrial expression of *FABP3* gene and autophagy-related genes in chronic AF patients may support our hypothesis that in chronic AF, autophagy at least in part contributes to the prevention of the accumulation of toxic fatty acid metabolites via a degradation of intracellular lipids. Further research is necessary to clarify the mechanistic roles of cardiac autophagy in the atrium in AF progression.

### The difference in the atrial expression of FABP3 between post-operative AF and chronic AF

We observed that the atrial gene expression of *FABP3* was reduced in patients with post-operative AF in our prior study [16]; this is inconsistent with our present results regarding chronic AF patients. One of the possible explanations is a difference in pathophysiology between post-operative AF and chronic AF. It was demonstrated that in patients with metabolic syndrome, impairment in the mitochondrial respiratory capacity in the atrial tissues predicts the occurrence of post-operative AF [28]. In contrast, the mitochondrial respiratory capacity in the atrium was reported to be increased in chronic AF patients [29]. Taken together, these findings indicate that impaired energy metabolism (including reduced fatty acid utilization) in the atrial muscle might be a primary pathogenesis of post-operative AF, but in chronic AF, excessive fatty acid uptake into the atrial cells seems to play a crucial role in AF progression.

### Study limitations

Several limitations of this study should be addressed. First, most of the patients had valvular heart diseases, and the results of this study thus cannot be directly applied to patients with lone AF. Second, the number of AF patients was smaller than that of SR patients. Third, we cannot completely exclude the possibility of effects of medicine including diuretics and beta-blockers on fatty acid metabolism in chronic AF patients. Finally, we cannot conclude that there is a causal relationship between fatty acid metabolism and autophagy in the atrium.

## Conclusions

Our study is the first to demonstrate that compared to patients with SR, the atrial expression of *FABP3* gene was upregulated in association with autophagy-related genes in patients with chronic AF. We also observed that the atrial gene expression of *FABP3* was related to structural and electrical remodeling in SR patients. Despite the increased atrial expression of *FABP3* with higher serum levels of FFA, atrial contents of DAG were not increased in patients with chronic AF. These findings provide new insights into the pathophysiology of chronic AF, and they suggest that dysregulated cardiac fatty acid metabolism might contribute to the progression of AF and induction of autophagy might have a cardioprotective effect against cardiac lipotoxicity in chronic AF.

## Supporting information

S1 Table Blood biochemistry measurements. (DOCX)

S2 Table Probes and primers of genes. (DOCX)
