# Supplementary material for: Correlation between increased atrial expression of genes related to fatty acid metabolism and autophagy in patients with chronic atrial fibrillation
Source: PLoS One. 2020 Apr 21;15(4):e0224713. doi: 10.1371/journal.pone.0224713 (PMC7173849; doi:10.1371/journal.pone.0224713)
Supplement: S1 Table — (DOCX) [file pone.0224713.s001.docx]

|  | **Method** | **Analyzer** |
| --- | --- | --- |
| Glucose | Enzymatic reaction involving glucose oxidase | GA08II (A&T, Yokotahama, Japan) |
| HbA1c | High-performance liquid chromatography | HLC-723G8 (Tosoh, Tokyo, Japan) |
| Insulin | One-step sandwich enzyme-linked immunosorbent assay | AIA-1800 (Tosoh, Tokyo, Japan) |
| FFA | Enzymatic method | BioMajesty JCA-BM8060 (JEOL, Akishima, Japan) |
| Total cholesterol | Enzymatic method | JCA-BM8030 (JEOL, Tokyo, Japan) |
| Triglycerides | Enzymatic method | JCA-BM8031 (JEOL, Tokyo, Japan) |
| BNP | Chemiluminescence immunoassay | ARCHITECT i2000 (Abbott Laboratories, Abbott Park, Ill) |

**Supplementary Table 1. Blood biochemistry measurements.**

BNP, B-type natriuretic peptide; FFA, free fatty acid; HbA1c, hemoglobin A1c.
