# Supplementary material for: Correlation between increased atrial expression of genes related to fatty acid metabolism and autophagy in patients with chronic atrial fibrillation
Source: PLoS One. 2020 Apr 21;15(4):e0224713. doi: 10.1371/journal.pone.0224713 (PMC7173849; doi:10.1371/journal.pone.0224713)
Supplement: S2 Table — (DOCX) [file pone.0224713.s002.docx]

|  | **UPL Cat.No.** | **UPL probe number** | **Forward primer** | **Reverse primer** |
| --- | --- | --- | --- | --- |
| CD36 | 04692152001 | 98 | TTATCCAGAAGACAATTAAAAAGCAA | GCACATCAAAGATCCAAAACTG |
| CPT1B | 04685091001 | 10 | GAGCAGCACCCCAATCAC | AACTCCATAGCCATCATCTGCT |
| FABP3 | 04688074001 | 47 | AGCAGATGACAGGAAGGTCAA | TCTGCAGGTGAACAAGTTTCC |
| ATG5 | 04694333001 | 147 | GGATGGGATTGCAAAATGAC | TCTGCAGGATATTCCATGAGTTT |
| ULK1 | 04688651001 | 66 | CATCGTCTACCAGTGCCTGA | GGGACCAACGTCTTGTTCTT |
| BECN1 | 04686934001 | 20 | GGATGGTGTCTCTCGCAGAT | TTGGCACTTTCTGTGGACAT |
| LC3 | 04694392001 | 153 | CGGACATCTACGAGCAGGAG | TTTCCTGGGAGGCGTAGAC |
| TBP | 04692250001 | 106 | GTGCTCACCCACCAACAAT | GGAGAACAATTCTGGGTTTGA |

**Supplementary Table 2. Probes and primers of genes.**

ATG5, autophagy-related gene 5; BCLN1, Beclin-1; CD36, cluster of differentiation 36/fatty acid translocase; CPT1B, carnitine palmitoyltransferase 1B; FABP3, fatty acid-binding protein 3; LC3, microtubule-associated protein light chain 3; TBP, TATA-binding protein; ULK1, Unc-51-like kinase 1; UPL, universal probe library
